# Structure-Based Discovery of a Series of 5*H*-Pyrrolo[2,3-*b*]pyrazine FGFR Kinase Inhibitors

**DOI:** 10.3390/molecules23030698

**Published:** 2018-03-19

**Authors:** Alan Jiang, Qiufeng Liu, Ruifeng Wang, Peng Wei, Yang Dai, Xin Wang, Yechun Xu, Yuchi Ma, Jing Ai, Jingkang Shen, Jian Ding, Bing Xiong

**Affiliations:** 1 College of Pharmacy, Nanchang University, 461 Bayi Avenue, Nanchang 330006, China; aljiang@jding.dhs.org (A.J.); jding@simm.ac.cn (J.D.); 2 Division of Anti-Tumor Pharmacology, State Key Laboratory of Drug Research, Shanghai Institute of MateriaMedica, Chinese Academy of Sciences, 555 Zuchongzhi Road, Shanghai 201203, China; daiyang@simm.ac.cn; 3 Drug Design and Discovery Center, Shanghai Institute of Materia Medica, Chinese Academy of Sciences, 555 Zuchongzhi Road, Shanghai 201203, China; lauraove2012@simm.ac.cn; 4 Key Laboratory of Structure-Based Drug Design and Discovery of Ministry of Education, Shenyang Pharmaceutical University, Shenyang 110016, China; rfwang1992@126.com (R.W.); weip1993@163.com (P.W.); 5 Department of Medicinal Chemistry, Shanghai Institute of Materia Medica, Chinese Academy of Sciences, 555 Zuchongzhi Road, Shanghai 201203, China; wangxin@simm.ac.cn (X.W.); jkshen@simm.ac.cn (J.S.)

**Keywords:** cancer, FGFR, kinase inhibitor, pyrrolo[2,3-*b*]pyrazine

## Abstract

Fibroblast growth factor receptors (FGFRs), a subfamily of receptor tyrosine kinases, are aberrant in various cancer types, and considered to be promising targets for cancer therapy. We started with a weak-active compound that was identified from our internal hepatocyte growth factor receptor (also called c-Met) inhibitor project, and optimized it with the guidance of a co-crystal structure of compound 8 with FGFR1. Through rational design, synthesis, and the biological evaluation of a series of 5*H*-pyrrolo[2,3-*b*]pyrazine derivatives, we discovered several potent FGFR kinase inhibitors. Among them, compound 13 displayed high selectivity and favorable metabolic properties, demonstrating a promising lead for further development.

## 1. Introduction

**Figure 1:**
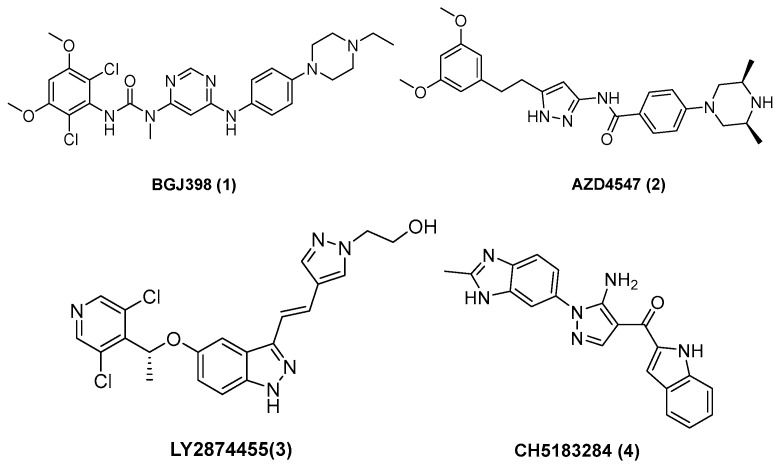
Representative selective fibroblast growth factor receptor (FGFR) inhibitors.

Fibroblast growth factors receptors (FGFR1-4) are a subfamily of receptor tyrosine kinases (RTKs) that are involved in many cellular processes such as angiogenesis, embryogenesis, tissue homeostasis, wound repair, and cancer [1,2,3]. In the human genome, 22 fibroblast growth factors (FGFs) have been identified as ligands of FGFRs, and are usually classified into seven subfamilies, according to their biochemical function sequence similarity and evolutionary relationships [4,5]. Upon binding with fibroblast growth factors, FGFRs induce dimerization and autophosphorylation on tyrosine residues, resulting in the activation of kinases downstream signaling, including MEK-ERK, PI3K-Akt, and PLCγ [6,7]. Numerous evidences highlight that the activation of FGF/FGFR signaling plays a critical role in tumor progression and growth [8,9,10]. Moreover, aberrant signaling of FGF/FGFR has been frequently found in various cancers, making FGFR a hot therapeutic target in anticancer drug development [11]. Selective and potent FGFR inhibitors are needed, because, in general, compounds that are selective to one intended target kinase can potentially claim a more favorable safety profile than multi-target compounds [12,13,14]. Currently, several FGFR-targeted small molecules have been evaluated in clinical trials for cancer treatment, such as NVP-BGJ398 (**1**) [15], AZD4547 (**2**) [16,17], LY2874455 (**3**) [18], and CH5183284 (**4**) [19] (Figure 1). Those FGFR inhibitors exhibited distinct effects on different mutants, as they may provide unique therapeutic benefits for certain patients, so that developing FGFR inhibitors with a novel scaffold is in constant demand.

**Figure 2:**
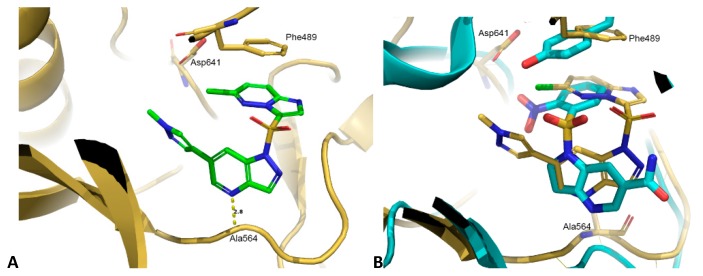
(**A**) The crystal structure of compound **8** bound to FGFR1 (PDB ID: 5Z0S). (**B**) Superimposed the crystal structure of a sulfoamide ligand bound to a hepatocyte growth factor receptor (c-Met) (PDB ID: 2WDI) to the structure of compound **8** bound to FGFR1. The protein was depicted in cartoon style, while the inhibitors and interacted residues were shown as sticks.

Previously, we described a series of 1-sulfonylpyrazolo[4,3-*b*]pyridines as potent and selective hepatocyte growth factor receptor (c-Met) inhibitors [20]. In the development of c-Met kinase inhibitors, we found that several 1-sulfonylpyrazolo[4,3-*b*]pyridines showed definite activity against FGFR1 at 10 μM, as shown in Table 1. Since the 1-sulfonylpyrazolo[4,3-*b*]pyridines as selective c-Met inhibitors have been reported [20], we are inquisitive about the binding mode of this chemotype as FGFR inhibitors. Therefore, we solved the X-ray crystal structure (PDB ID: 5Z0S) of compound **8** with FGFR1, as illustrated in Figure 2. Interestingly, the co-crystal structure of compound **8** bound to the FGFR1 kinase domain revealed novel binding interactions, which has not been reported. In this paper, we present our structure optimization of this series of compounds as FGFR inhibitors, in the hope that the investigation can stimulate new ideas for developing selective FGFR inhibitors as anticancer drugs.

## 2. Results and Discussion

The biological activity of compounds (**5**, **6**, **7**, **8**) as c-Met inhibitors was reported in our previous work [20], the compounds with the scaffold of 1-sulfonylpyrazolo[4,3-*b*]pyridines showed definite FGFR1 activity at 10 μM, all with a more than 50% inhibition ratio towards FGFR1 (Table 1). The X-ray crystal structure of compound **8** with FGFR1 revealed that the pyrazolo[4,3-*b*]pyridine scaffold forms a hydrogen bond, with the backbone of residue Ala564 at the hinge part. The P-loop of FGFR1 lowered down to form a pi–pi stacking interaction between residue Phe489 and the imidazo[1,2-*b*]pyridazine ring. Although these interactions similarly existed in the co-crystal structure of c-Met bound with inhibitors (PDB ID: 2WDI), the most striking difference is that the overall binding conformation of inhibitor **8** is inversed, giving that the methylpyrazole pointed towards the back pocket of the ATP site of FGFR1, which can be immediately noticed from the superimposed structures, as shown in Figure 2B. Due to the novel binding interactions of compound **8**, we thought it should be possible to selectively optimize the FGFR activity of this series of compounds. Based on our previous study, changing the scaffold of 1*H*-pyrazolo[4,3-*b*]pyridine to 5*H*-pyrrolo[2,3-*b*]pyrazine can increase the binding activity of FGFR1 [21]; therefore, we synthesized the compounds **9** and **10**. As listed in Table 2, the activity of compounds **9** and **10** has increased slightly compared with compounds **5** and **6**, respectively. In addition, the scaffold of 5*H*-pyrrolo[2,3-*b*]pyrazine also has been reported to have Bruton’s tyrosine kinase, focal adhesion kinase, JAK3, ataxia telangiectasia and Rad3-related protein (ATR), and serine/threonine kinase activity [22,23,24,25,26,27]. Therefore, we selected 5*H*-pyrrolo[2,3-*b*]pyrazine as the starting scaffold for further modification.

It is apparently noticed from compounds **5**, **7**, **8**, and **9** that they all contained an imidazole ring that formed the π–π interaction with the residue Phe489. Therefore, we attempted to simplify the bicycle to imidazole so that compound **11** was synthesized, which maintained the activity against FGFR1, as shown in Table 2. Furthermore, substituting the methyl of imidazole to 2-fluoroethyl (**12**) could slightly increase the inhibitory activity. Based on the advantage of synthesis, we selected compound **12** as the starting point for further modification.

Based on the X-ray structure of compound **8** bound to FGFR1, the 1-methyl-1*H*-pyrazole of compound **8** extended to the inside of the ATP site of FGFR1, where there are a large number of polar residues and a corresponding cavity for optimization. In order to investigate the structure-activity relationship (SAR) around the methylpyrazole part, we synthesized **13**–**26** by substituting the methyl group with different groups, and their FGFR1 enzymatic activities are summarized in Table 3. Interestingly, the compound **13**, which bears an unsubstituted pyrazole ring, showed much higher activity than the others, and even at 10 nM concentration, it still has a more than 90% inhibition ratio (IC_50_ = 0.6 nM, as shown in Table 5). When isopropyl was induced to the structure, the compound (**14**) lost the FGFR1 activity. Compounds **15**–**17**, containing a carbonyl group as hydrogen bond receptors, have moderate activity. Meanwhile, for the compounds with carbonyl groups that are deeper into the protein cavity (**18**–**22**), neither ester (**19**, **20**) nor acylamido (**21**, **22**) showed weaker activity than compound **15**. Compared with the carbonyl group, the mesyl (**23**) caused a slight increase in inhibitory activity; however, replacing the mesyl with a larger cyclopropylsulfonyl group (**24**) decreased the inhibitory activity. Doubling the carbonyl group (**25**) showed stronger activity than monocarbonyl (**15**), which may be due to forming more hydrogen bonds. In summary, all of the attempts at optimizing the substitutes on pyrazole couldn’t significantly improve the activity of FGFR1; therefore, we further carried out the optimization by focusing on compound **13**.

**Figure 3:**
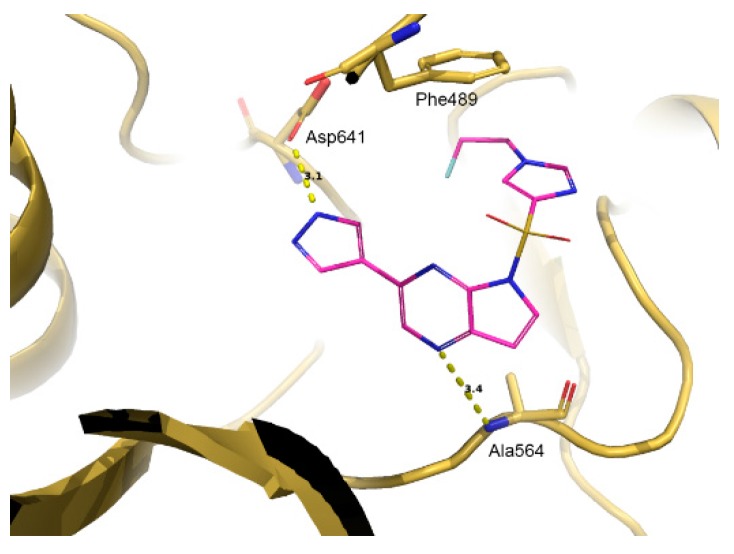
The docking study of compound **13** to FGFR1, and the model was prepared from the co-crystal structure of compound **8** with FGFR1 (PDB ID: 5Z0S).

To find the mechanism of this significant activity improvement of compound **13**, we adopted the docking method to predict the binding interactions between compound **13** and FGFR1. As illustrated in Figure 3, compound **13** bound to FGFR1 with a very similar mode to how compound **8** bound, by using the nitrogen atom in the pyrazine ring to form an essential hydrogen bond with the FGFR1 hinge and using imidazole stacking with the residue Phe489. Besides, compound **13** also formed a salt bridge interaction between the pyrazole and Asp641. We assumed this interaction dramatically increased the binding affinity of compound **13**.

We further optimized the right side of compound **13**. The designed and synthesized compounds **27**–**35** with modifications on the imidazole moiety of compound **13** were assessed for their FGFR1 inhibitory activities (Table 4). Firstly, substituents from small to large groups, namely methyl (**28**), ethyl (**29**), isopropyl (**30**), isobutyl (**31**), and cyclopentylmethyl (**32**), were induced to imidazole. The results indicated that the substitution of the imidazole ring with ethyl (**29**, FGFR1 IC_50_ = 3.0 nM) and isopropyl (**30**, FGFR1 IC_50_ = 3.0 nM) showed better inhibitory activity among the compounds (**27**–**32)**. Incorporation of the methylacetamide (**33**) group reduced the enzymatic potency. The activity of compounds **34** and **35** showed that the bicycles were weaker than the imidazole.

Based on the structure diversity and FGFR1 enzymatic inhibition ratio, we selected four compounds to obtain the accurate IC_50_ values and assess their antiproliferation activity In KG1 cells. As listed in Table 5, compounds **13** and **29** showed excellent biological activities. Since this series of compounds stemmed from our c-Met inhibitors, compound **13** was selected as the representative to test 17 kinases’ (include c-Met) enzymatic activity. As shown in Table 6, compound **13** demonstrated nearly no inhibition towards c-Met, even at a concentration of 1 μM, indicating that compound **13** may be a selective FGFR inhibitor.

To further assess the druggability of compound **13**, it was further subjected to in vitro metabolic stability assays and an in vivo pharmacokinetic test. Compound **13** showed excellent metabolic stability, as it has a low clearance ratio of 1 μL/min/mg in the human liver microsome. In addition to the liver microsome assay, five human cytochrome P450 (CYP) enzymes commonly metabolizing exogenous chemicals were used to test the direct inhibition of compound **13**. As shown in Table 7, the compound showed favorable metabolic properties, as the inhibition ratio for five CYPs were less than 50%, even at the compound concentration of 10 μM.

## 3. Materials and Methods

### 3.1. Chemistry

**Scheme 1:**
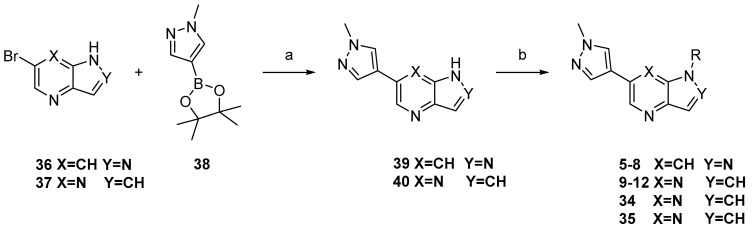
Reagents and conditions: (a) Pd(dppf)Cl_2_, K_2_CO_3_, Dioxane: H_2_O (*v*/*v* = 3:1), 80 °C, 2 h, 82% yield (**39**), 86% yield (**40**); (b) NaH, Dimethylformamide, R-SO_2_Cl, r.t., 1–4 h, 76–83% yield.

Compounds **5**–**12**, **34**, and **35** were synthesized according to the procedures outlined in Scheme 1. A Suzuki coupling of commercially available **36** and **37** with 1-methylpyrazole-4-boronic acid pinacol ester (**38**) provided **39** and **40**, respectively**.** Compounds **5**–**12**, **34**, and **35** were prepared by the deprotonation of compound **39** or **40** deprotonation, followed by the addition of the corresponding electrophile reagents.

**Scheme 2:**
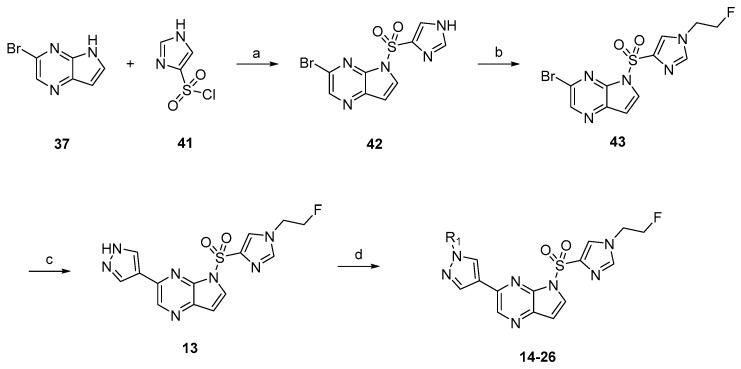
Reagents and conditions: (a) NaH, Dimethylformamide, r.t., 4 h, 84% yield; (b) NaH, Dimethylformamide, 1-fluoro-2-iodoethane, 80 °C, 4 h, 90% yield; (c) 4-(4,4,5,5-tetramethyl-1,3,2-dioxaborolan-2-yl)-1*H*-pyrazole, K_2_CO_3_, Pd(PPh_3_)Cl_2_, Dioxane: H_2_O (*v:v* = 3:1), 80 °C, 4 h, 87% yield; (d) K_2_CO_3_, Dimethylformamide, r.t., 8 h, 70–89% yield.

Compounds **13**–**26** were prepared according to the procedure in Scheme 2. Compound **42** was prepared by the deprotonation of compound **37** followed by addition of the 1*H*-imidazole-4-sulfonyl chloride (**41**). Compound **43** was synthesized via substitution by 1-fluoro-2-iodoethane from compound **42**. A Suzuki coupling of compound **43** with 1*H*-pyrazole-4-boronic acid pinacol ester provided compound **13**. A variety of groups were introduced at the 1-position of pyrazole of compound **13** via a substituent reaction to provide **14**–**26**. Compounds **44**–**50** were also generated by substituent reaction from compound **42**; then, 1*H*-pyrazole-4-yl was introduced at the 3-position of the corresponding compounds (**44**–**50**) via a Suzuki coupling reaction to provide **27**–**33**.

**Scheme 3:**
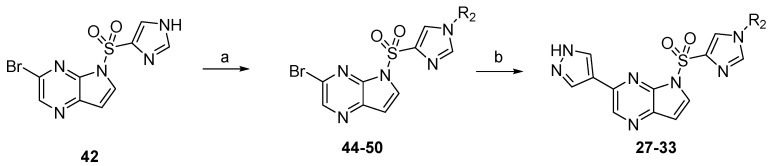
Reagents and conditions: (a) NaH, Dimethylformamide, 80 °C, 4 h, 70–89% yield; (b) 4-(4,4,5,5-tetramethyl-1,3,2-dioxaborolan-2-yl)-1*H*-pyrazole, K_2_CO_3_, Pd(PPh_3_)Cl_2_, Dioxane: H_2_O (*v*:*v* = 3:1), 80 °C, 4 h, 76–84% yield.

Compounds **27**–**33** were prepared according to the procedure in Scheme 3. Compounds **44**–**50** were also generated by a substituent reaction from compound **42**; then, 1*H*-pyrazole-4-yl was introduced at the 3-position of the corresponding compounds (**44**–**50**) via a Suzuki coupling reaction to provide compounds **27**–**33**.

#### 3.1.1. General Methods of Chemistry

All of the melting points of the compounds were determined on a micro melting point apparatus, and were uncorrected. ^1^H-NMR and ^13^C-NMR spectra were obtained via a Bruker Avance-400 NMR-spectrometer. Chemical shifts were expressed in δ units, and Tetramethylsilane (TMS) as an internal reference. Mass spectra was conducted via a LC Autosampler Device: Standard G1313A instrument. The purity of all of the compounds was determined by analytical Gilson high-performance liquid chromatography (HPLC) using an YMC ODS3 column (50 mm × 4.6 mm, 5 ZM). Conditions were as follows: CH_3_CN/H_2_O eluent at 2.5 mL·min^−1^ flow [containing 0.1% trifluoroacetic acid (TFA)] at 35 °C, 8 min, gradient 5% CH_3_CN to 95% CH_3_CN, monitored by UV absorption at 214 nm and 254 nm. The purity of new compounds in this paper is only assessed by HPLC data. Flash column chromatography was performed on a column packed with Silica Gel 60 (200–300 mesh). Meanwhile, Thin-layer chromatography (TLC) was taken on Silica Gel GF254 for TLC, and spots were visualized by irradiation with UV light (λ = 254 nm). Solvents were of reagent grade, which were purified and dried by standard methods when necessary. The concentration of the reaction solutions used the rotary evaporator under reduced pressure conditions. 

General Procedure for the Synthesis of 6-(1-methyl-1*H*-pyrazol-4-yl)-1*H*-pyrazolo[4,3-*b*]pyridine (**39**). 6-bromo-1*H*-pyrazolo[4,3-*b*]pyridine (400 mg, 2.0 mmol), 1-methyl-4-(4,4,5,5-tetramethyl-1,3,2-dioxaborolan-2-yl)-1*H*-pyrazole (500 mg, 2.4 mmol), K_2_CO_3_ (828 mg, 6 mmol), and Pd(dppf)Cl_2_ (81.7 mg, 0.1 mmol) were dissolved in the solvent of 1,4-dioxane/H_2_O (*v*/*v* = 3:1, 20 mL). The resulting mixture was stirred at 80 °C under Ar for 2 h. Then, the reaction mixture was evaporated to dryness. The residue was purified by flash chromatography to give compound **39** as a yellow hairy solid with the yield of 82%. ^1^H-NMR (300 MHz, DMSO-*d*_6_, ppm) δ 13.29 (s, 1H), 8.80 (s, 1H), 8.36 (s, 1H), 8.24 (s, 1H), 8.07 (s, 2H), 3.90 (s, 3H). ESI-MS: C_10_H_10_N_7_O_2_S, Exact Mass: 199.09, *m*/*z* 200.0 (M + 1)^+^. Retention time 2.51 min, >95% purity.

#### 3.1.2. Compound **40** Was Prepared as Described for the Synthesis of Compound **39**

*3-(1-methyl-1H-pyrazol-4-yl)-5H-pyrrolo[2,3-b]pyrazine* (**40**). Yield: 86%; yellow solid. ^1^H-NMR (400 MHz, DMSO-*d*_6_) δ 9.30 (s, 1H), 8.72 (s, 1H), 8.11 (s, 1H), 7.97 (s, 1H), 7.28 (s, 1H), 6.74 (dd, *J* = 3.7, 1.9 Hz, 1H), 4.02 (s, 3H). ESI-MS: C_10_H_9_N_5_, Exact Mass: 199.09, *m*/*z* 200.1 (M + 1)^+^. Retention time 2.56 min, >98% purity.

General Procedure for the Synthesis of *6-chloro-5-((6-(1-methyl-1H-pyrazol-4-yl)-1H-pyrazolo[4,3-b]pyridin-1-yl)sulfonyl)imidazo[2,1-b]thiazole* (**5**). NaH (6.7 mg, 0.28 mmol) was suspended in dry DMF. 6-(1-methyl-1*H*-pyrazol-4-yl)-1*H*-pyrazolo[4,3-*b*]pyridine (**39**, 50 mg, 0.25 mmol) was added into the above solution slowly at 0 °C. Then, the cooling solution was stirred for 0.5 h. Subsequently, 6-chloroimidazo[2,1-*b*]thiazole-5-sulfonyl chloride (77 mg, 0.3 mmol) was added and stirred for another 4 h at room temperature. Then, water (30 mL) was added to the reaction system. The reaction mixture was extracted with ethyl acetate (3 × 40 mL). The combined organic phase was washed with saturated salt water (3 × 40 mL), dried over anhydrous Na_2_SO_4_, filtered, and concentrated under reduced pressure to give the corresponding crude target product, which was purified by flash column chromatography with dichloromethane/methanol to afford compound **5** as a white hairy solid with the yield of 78%. ^1^H-NMR (400 MHz, CDCl_3_, ppm) δ 8.88 (d, *J* = 1.8 Hz, 1H), 8.54 (s, 1H), 8.35 (s, 1H), 8.21 (d, *J* = 4.6 Hz, 1H), 7.93 (s, 1H), 7.84 (s, 1H), 7.17 (d, *J* = 4.5 Hz, 1H), 4.03 (s, 3H). ^13^C-NMR (126 MHz, CDCl_3_) δ 151.78, 147.08, 142.45, 141.49, 140.84, 137.30, 134.38, 129.09, 128.07, 121.12, 119.20, 116.39, 116.32, 115.13, 39.41. ESI-MS: C_15_H_10_ClN_7_O_2_S_2_, Exact Mass: 419.0, *m*/*z* 420.0 (M + 1)^+^. HRMS-ESI *m*/*z* calcd. for C_15_H_11_ClN_7_O_2_S_2_ [M + H]^+^ 420.0099, found 420.0135. Retention time 2.53 min, >98% purity.

The compounds **6**–**12**, **34**, **35** were prepared as described for the synthesis of compound **5** (Scheme 1).

*6-(1-Methyl-1H-pyrazol-4-yl)-1-(pyrazolo[1,5-a]pyrimidin-3-ylsulfonyl)-1H-pyrazolo[4,3-b]pyridine* (**6**). Yield: 79%; white solid. ^1^H-NMR (400 MHz, CDCl_3_, ppm) δ 8.83 (d, *J* = 1.8 Hz, 1H), 8.76 (dd, *J* = 7.0, 1.7 Hz, 1H), 8.71 (d, *J* = 4.1 Hz, 2H), 8.66 (s, 1H), 8.35 (s, 1H), 7.97 (s, 1H), 7.88 (s, 1H), 7.11 (dd, *J* = 6.9, 4.3 Hz, 1H), 4.03 (s, 3H). ^13^C-NMR (126 MHz, DMSO-*d*_6_) δ 155.14, 146.36, 146.16, 145.60, 141.25, 140.73, 138.19, 136.83, 133.71, 129.34, 128.34, 118.21, 115.37, 112.00, 105.05, 38.81. ESI-MS: C_16_H_13_N_8_O_2_S, Exact Mass: 380.08, *m*/*z* 381 (M + 1)^+^. HRMS-ESI *m*/*z* calcd. for C_16_H_13_N_8_O_2_S [M+H]^+^ 381.0877, found 381.0891. Retention time 2.55 min, >98% purity.

*1-(Imidazo[1,2-b]pyridazin-3-ylsulfonyl)-6-(1-methyl-1H-pyrazol-4-yl)-1H-pyrazolo[4,3-b]pyridine* (**7**). Yield: 80%; yellow solid. ^1^H-NMR (400 MHz, CDCl_3_, ppm) δ 8.87 (d, *J* = 1.9 Hz, 1H), 8.64 (dd, *J* = 1.9, 0.9 Hz, 1H), 8.58 (s, 1H), 8.35 (d, *J* = 0.9 Hz, 1H), 8.30 (dd, *J* = 4.6, 1.6 Hz, 1H), 8.07 (dd, *J* = 9.3, 1.6 Hz, 1H), 7.98 (d, *J* = 0.8 Hz, 1H), 7.94–7.87 (m, 1H), 7.26–7.22 (m, 1H), 4.04 (s, 3H). ^13^C-NMR (126 MHz, DMSO-*d*_6_, ppm) δ 146.58, 146.08, 142.87, 142.14, 141.13, 140.46, 136.89, 134.52, 129.44, 128.66, 126.90, 122.26, 121.22, 118.12, 115.41, 38.81. ESI-MS: C_16_H_13_N_8_O_2_S, Exact Mass: 380.08, *m*/*z* 381.08 (M + 1)^+^. HRMS-ESI *m*/*z* calcd. for C_16_H_13_N_8_O_2_S [M + H]^+^ 381.0877, found 381.0974. Retention time 2.60 min, >98% purity.

*1-((6-Bromoimidazo[1,2-a]pyridin-3-yl)sulfonyl)-6-(1-methyl-1H-pyrazol-4-yl)-1H-pyrazolo[4,3-b]pyridine* (**8**). Yield: 78%; white solid. ^1^H-NMR (400 MHz, CDCl_3_, ppm) δ 8.90 (d, *J* = 1.9 Hz, 1H), 8.73 (d, *J* = 1.0 Hz, 1H), 8.56 (s, 1H), 8.41–8.36 (m, 1H), 8.05–7.98 (m, 2H), 7.93 (s, 1H), 7.22 (d, *J* = 9.6 Hz, 1H), 4.05 (s, 3H). ESI-MS: C_16_H_11_ClN_8_O_2_S, Exact Mass: 414.04, *m*/*z* 415.10 (M + 1)^+^. HRMS-ESI *m*/*z* calcd. for C_16_H_12_ClN_8_O_2_S [M + H]^+^ 415.0487, found 415.0555. 

*6-Chloro-5-((3-(1-methyl-1H-pyrazol-4-yl)-5H-pyrrolo[2,3-b]pyrazin-5-yl)sulfonyl)imidazo[2,1-b]thiazole* (**9**). Yield: 80%; white solid. ^1^H-NMR (400 MHz, CDCl_3_, ppm) δ 8.68 (s, 1H), 8.42 (d, *J* = 4.5 Hz, 1H), 8.02 (d, *J* = 4.1 Hz, 1H), 7.78 (d, *J* = 9.0 Hz, 2H), 7.23 (d, *J* = 4.6 Hz, 1H), 6.83 (d, *J* = 4.1 Hz, 1H), 3.97 (s, 3H). ESI-MS: C_15_H_10_ClN_7_O_2_S_2_, Exact Mass: 419.00, *m*/*z* 420.02 (M + 1)^+^. HRMS-ESI *m*/*z* calcd. for C_15_H_11_ClN_7_O_2_S_2_ [M + H]^+^ 420.0099, found 420.0121. Retention time 2.58 min, >98% purity.

*3-(1-Methyl-1H-pyrazol-4-yl)-5-(pyrazolo[1,5-a]pyrimidin-3-ylsulfonyl)-5H-pyrrolo[2,3-b]pyrazine* (**10**). Yield: 79%; pale white solid. ^1^H-NMR (400 MHz, CDCl_3_, ppm) δ 8.76 (s, 1H), 8.67 (s, 1H), 8.50 (dd, *J* = 4.4, 1.4 Hz, 1H), 8.17 (d, *J* = 4.1 Hz, 1H), 8.07 (dd, *J* = 9.2, 1.4 Hz, 1H), 7.96 (d, *J* = 11.6 Hz, 2H), 7.33–7.27 (m, 1H), 6.81 (d, *J* = 4.2 Hz, 1H), 4.01 (s, 3H). ESI-MS: C_16_H_12_N_8_O_2_S, Exact Mass: 380.08, *m*/*z* 381.05 (M + 1)^+^. HRMS-ESI *m*/*z* calcd. for C_16_H_13_N_8_O_2_S [M + H]^+^ 381.0877, found 381.0976. Retention time 2.67 min, >98% purity.

*5-((1-Methyl-1H-imidazol-4-yl)sulfonyl)-3-(1-methyl-1H-pyrazol-4-yl)-5H-pyrrolo[2,3-b]pyrazine* (**11**). Yield: 75%; white solid. ^1^H-NMR (400 MHz, CDCl_3_, ppm) δ 8.71 (s, 1H), 8.06 (s, 1H), 8.04–8.02 (m, 1H), 8.01 (s, 1H), 7.90 (s, 1H), 7.43 (d, *J* = 1.3 Hz, 1H), 6.82 (d, *J* = 4.1 Hz, 1H), 4.02 (s, 3H), 3.75 (s, 3H). ESI-MS: C_14_H_13_N_7_O_2_S, Exact Mass: 343.09, *m*/*z* 343.07 (M + 1)^+^. HRMS-ESI *m*/*z* calcd. for C_14_H_14_N_7_O_2_S [M + H]^+^ 344.0924, found 344.1037. Retention time 2.60 min, >98% purity.

*5-((1-(2-Fluoroethyl)-1H-imidazol-4-yl)sulfonyl)-3-(1-methyl-1H-pyrazol-4-yl)-5H-pyrrolo[2,3-b]pyrazine* (**12**). Yield: 85%; yellow solid. ^1^H-NMR (400 MHz, DMSO-*d*_6_, ppm) δ 8.92 (s, 1H), 8.66 (s, 1H), 8.41 (s, 1H), 8.14 (s, 1H), 8.10 (d, *J* = 4.1 Hz, 1H), 7.86 (s, 1H), 6.93 (d, *J* = 4.0 Hz, 1H), 4.77 (t, *J* = 4.8 Hz, 1H), 4.65 (t, *J* = 4.6 Hz, 1H), 4.43 (t, *J* = 4.5 Hz, 1H), 4.36 (t, *J* = 4.7 Hz, 1H), 3.92 (s, 3H). ESI-MS: C_15_H_15_FN_7_O_2_S, Exact Mass: 375.09, *m*/*z* 376.15 (M + 1)^+^. HRMS-ESI *m*/*z* calcd. for C_15_H_15_FN_7_O_2_S [M + H]^+^ 376.0986, found 376.1195. Retention time 2.51 min, >98% purity.

*3-(1H-Pyrazol-4-yl)-5-(pyrazolo[1,5-a]pyrimidin-3-ylsulfonyl)-5H-pyrrolo[2,3-b]pyrazine* (**34**). Yield: 81%; white solid. ^1^H-NMR (400 MHz, DMSO-*d*_6_, ppm) δ 13.24 (s, 1H), 9.03 (s, 1H), 8.93 (s, 1H), 8.71 (dd, *J* = 4.5, 1.5 Hz, 1H), 8.44 (s, 1H), 8.34 (dd, *J* = 9.3, 1.5 Hz, 1H), 8.27 (d, *J* = 4.2 Hz, 1H), 8.12 (s, 1H), 7.51 (dd, *J* = 9.3, 4.6 Hz, 1H), 6.97 (d, *J* = 4.2 Hz, 1H). ESI-MS: C_15_H_11_N_8_O_2_S, Exact Mass: 366.06, *m*/*z* 367.08 (M + 1)^+^. HRMS-ESI *m*/*z* calcd. for C_15_H_11_N_8_O_2_S [M + H]^+^ 367.0720, found 367.0820. Retention time 2.47 min, >98% purity.

*5-(Imidazo[1,2-b]pyridazin-3-ylsulfonyl)-3-(1H-pyrazol-4-yl)-5H-pyrrolo[2,3-b]pyrazine* (**35**). Yield: 79%; white solid. ^1^H-NMR (400 MHz, DMSO-*d*_6_, ppm) δ 13.25 (s, 1H), 9.32 (dd, *J* = 7.0, 1.7 Hz, 1H), 9.25 (s, 1H), 8.93 (s, 1H), 8.84 (dd, *J* = 4.3, 1.7 Hz, 1H), 8.51 (s, 1H), 8.21 (d, *J* = 4.1 Hz, 1H), 8.19 (s, 1H), 7.37 (dd, *J* = 7.0, 4.3 Hz, 1H), 6.90 (d, *J* = 4.1 Hz, 1H). ^13^C-NMR (126 MHz, DMSO-*d*_6_, ppm) δ 155.46, 148.14, 145.79, 142.58, 139.90, 138.68, 138.63, 138.60, 137.94, 130.35, 128.22, 119.61, 112.35, 105.88, 105.68. ESI-MS: C_15_H_11_N_8_O_2_S, Exact Mass: 366.06, *m*/*z* 367.06(M + 1)^+^. HRMS-ESI *m*/*z* calcd. for C_15_H_11_N_8_O_2_S [M + H]^+^ 367.0720, found 367.0791. Retention time 2.40 min, >90% purity.

General Procedure for the Synthesis of *(5-((1H-imidazol-4-yl)sulfonyl)-3-bromo-5H-pyrrolo[2,3-b]pyrazine)* (**42**). NaH (160 mg, 6.71 mmol) was suspended in dry DMF. 3-bromo-5*H*-pyrrolo[2,3-*b*]pyrazine (1.20 g, 6.1 mmol) was added into the above solution slowly at 0 °C. Then, the cooling solution was stirred for 0.5 h. Subsequently, 1*H*-imidazole-4-sulfonyl chloride (1.22 g, 7.32 mmol) was added and stirred for 4 h at room temperature. Then, water (50 mL) was added to the reaction system. The reaction mixture was extracted with dichloromethane (3 × 40 mL). The combined organic phase was concentrated under reduced pressure to give the crude target product, which was purified by flash column chromatography with dichloromethane/methanol to afford compound **42** as a white solid with the yield of 84%. ^1^H-NMR (400 MHz, CD_3_OD, ppm) δ 8.62 (s, 1H), 8.22 (d, *J* = 1.1 Hz, 1H), 8.16 (d, *J* = 4.2 Hz, 1H), 7.79 (d, *J* = 1.0 Hz, 1H), 6.88 (d, *J* = 4.1 Hz, 1H). ESI-MS: C_9_H_7_BrN_5_O_2_S, Exact Mass: 326.94, *m*/*z* 328.11 (M + 1)^+^. Retention time 2.42 min, >98% purity.

General Procedure for the Synthesis of *(3-bromo-5-((1-(2-fluoroethyl)-1H-imidazol-4-yl)sulfonyl)-5H-pyrrolo[2,3-b]pyrazine*) (**43**). NaH (130 mg, 5.52 mmol) was suspended in dry DMF. After 5-((1*H*-imidazol-4-yl)sulfonyl)-3-bromo-5*H*-pyrrolo[2,3-*b*]pyrazine (**42**, 1.5 g, 4.6 mmol) was added into the above solution, the resulting mixture was stirred at room temperature for 0.5 h. Subsequently, 1-fluoro-2-iodoethane (960 mg, 5.52 mmol) was added and stirred at 80 °C for another 4 h. Then, water (30 mL) was added to the reaction system. The reaction mixture was extracted with dichloromethane (3 × 40 mL). The combined organic phase was washed with saturated salt water (3 × 40 mL), dried over anhydrous Na_2_SO_4_, filtered, and concentrated under reduced pressure to give the corresponding crude target product, which was purified by flash column chromatography with dichloromethane/methanol to afford compound **43** as a white solid with the yield of 90%. ^1^H-NMR (400 MHz, DMSO-*d*_6_, ppm) δ 8.76 (s, 1H), 8.46 (s, 1H), 8.25 (d, *J* = 4.2 Hz, 1H), 7.89 (s, 1H), 7.04 (d, *J* = 4.2 Hz, 1H), 4.78 (t, *J* = 4.7 Hz, 1H), 4.67 (t, *J* = 4.7 Hz, 1H), 4.44 (t, *J* = 4.7 Hz, 1H), 4.37 (t, *J* = 4.7 Hz, 1H). ESI-MS: C_11_H_10_BrFN_5_O_2_S, Exact Mass: 372.96, *m*/*z* 374.17 (M + 1)^+^. Retention time 2.84 min, >97% purity.

General Procedure for the Synthesis of *(5-((1-(2-fluoroethyl)-1H-imidazol-4-yl)sulfonyl)-3-(1H-pyrazol-4-yl)-5H-pyrrolo[2,3-b]pyrazine)* (**13**). Bromo-5-((1-(2-fluoroethyl)-1*H*-imidazol-4-yl)sulfonyl)-5*H*-pyrrolo[2,3-*b*]pyrazine (**43**, 1.2 g, 3.2 mmol), 4-(4,4,5,5-tetramethyl-1,3,2-dioxaborolan-2-yl)-1*H*-pyrazole (745 mg, 3.84 mmol), K_2_CO_3_ (1.32 g, 9.6 mmol), and Pd(dppf)Cl_2_ (130 mg, 0.16 mmol) was dissolved in the solvent of 1,4-dioxane/H_2_O (*v*/*v* = 3:1, 20 mL). The resulting mixture was stirred at 80 °C under Ar for 4 h. Then, the reaction mixture was evaporated to dryness. The residue was purified by flash chromatography to give compound **13** as a yellow solid with the yield of 87%. ^1^H-NMR (400 MHz, DMSO-*d*_6_, ppm) δ 13.23 (s, 1H), 8.97 (s, 1H), 8.67 (d, *J* = 1.2 Hz, 1H), 8.48 (s, 1H), 8.19 (d, *J* = 1.3 Hz, 1H), 8.10 (d, *J* = 4.1 Hz, 1H), 7.86 (s, 1H), 6.93 (d, *J* = 4.1 Hz, 1H), 4.75 (t, *J* = 4.7 Hz, 1H), 4.64 (t, *J* = 4.6 Hz, 1H), 4.43 (t, *J* = 4.6 Hz, 1H), 4.36 (t, *J* = 4.7 Hz, 1H). ESI-MS: C_14_H_13_FN_7_O_2_S, Exact Mass: 361.08, *m*/*z* 362.24 (M + 1)^+^. HRMS-ESI *m*/*z* calcd. for C_14_H_13_FN_7_O_2_S [M + H]^+^ 362.0830, found 362.0905. Retention time 2.29 min, >99% purity.

General Procedure for the Synthesis of *(1-(4-(5-((1-(2-fluoroethyl)-1H-imidazol-4-yl)sulfonyl)-5H-pyrrolo[2,3-b]pyrazin-3-yl)-1H-pyrazol-1-yl)ethan-1-one)* (**15**). 5-((1-(2-fluoroethyl)-1*H*-imidazol-4-yl)sulfonyl)-3-(1*H*-pyrazol-4-yl)-5*H*-pyrrolo[2,3-*b*]pyrazine (**13**, 50 mg, 0.14 mmol), acetyl chloride (14 mg, 0.17 mmol), and K_2_CO_3_ (38 mg, 0.28 mmol) was dissolved in dry DMF. The resulting mixture was stirred at room temperature for 8 h. Then, water (30 mL) was added to the reaction system. The reaction mixture was extracted with ethyl acetate (3 × 40 mL). The combined organic phase was washed with saturated salt water (3 × 40 mL), dried over anhydrous Na_2_SO_4_, filtered, and concentrated under reduced pressure to give the corresponding crude target product, which was purified by flash column chromatography with dichloromethane/methanol to afford compound **15** as a white hairy solid with the yield of 89%. ^1^H-NMR (400 MHz, CDCl_3_, ppm) δ 8.79 (s, 1H), 8.69 (s, 1H), 8.29 (s, 1H), 8.11 (d, *J* = 4.1 Hz, 1H), 8.09 (s, 1H), 7.52 (s, 1H), 6.86 (d, *J* = 4.1 Hz, 1H), 4.71(t, *J* = 4.5 Hz, 1H), 4.59 (t, *J* = 4.6 Hz, 1H), 4.32(t, *J* = 4.6 Hz, 1H), 4.26 (t, *J* = 4.5 Hz, 1H), 4.15 (s, 3H). ESI-MS: C_16_H_15_FN_7_O_3_S, Exact Mass: 403.09, *m*/*z* 404.24 (M + 1)^+^. Retention time 2.65 min, >95% purity.

The compounds **14**–**26** were prepared as described for the synthesis of compound **15** (Scheme 2).

*3-(1-Cyclopropyl-1H-pyrazol-4-yl)-5-((1-(2-fluoroethyl)-1H-imidazol-4-yl)sulfonyl)-5H-pyrrolo[2,3-b]pyrazine* (**14**). Yield: 72%; white solid. ^1^H-NMR (400 MHz, DMSO-*d*_6_, ppm) δ 8.93 (s, 1H), 8.69 (s, 1H), 8.49 (s, 1H), 8.12–8.10 (m, 2H), 7.86 (s, 1H), 6.93 (d, *J* = 4.1 Hz, 1H), 4.77 (t, *J* = 4.6 Hz, 1H), 4.65 (t, *J* = 4.6 Hz, 1H), 4.43 (t, *J* = 4.7 Hz, 1H), 4.36 (t, *J* = 4.6 Hz, 1H), 3.82 (m, 1H), 1.15–1.10 (m, 2H), 1.05–1.02 (m, 2H). ESI-MS: C_17_H_17_FN_7_O_2_S, Exact Mass: 401.11, *m*/*z* 402.16 (M + 1)^+^. HRMS-ESI *m*/*z* calcd. for C_17_H_17_FN_7_O_2_S [M + H]^+^ 402.1143, found 402.1376. Retention time 2.76 min, >96% purity.

*1-(4-(5-((1-(2-Fluoroethyl)-1H-imidazol-4-yl)sulfonyl)-5H-pyrrolo[2,3-b]pyrazin-3-yl)-1H-pyrazol-1-yl)propan-1-one* (**16**). Yield: 83%; yellow solid. ^1^H-NMR (400 MHz, CDCl_3_, ppm) δ 8.79 (s, 1H), 8.74 (s, 1H), 8.24 (s, 1H), 8.12–8.08 (m, 2H), 7.52 (s, 1H), 6.85 (d, *J* = 4.1 Hz, 1H), 4.72 (t, *J* = 4.6 Hz, 1H), 4.61 (t, *J* = 4.5 Hz, 1H), 4.34 (t, *J* = 4.4 Hz, 1H), 4.28 (t, *J* = 4.6 Hz, 1H), 3.21 (q, *J* = 7.5 Hz, 2H), 1.33 (t, *J* = 7.3 Hz, 3H). ESI-MS: C_17_H_17_FN_7_O_3_S, Exact Mass: 417.10, *m*/*z* 418.05 (M + 1)^+^. HRMS-ESI *m*/*z* calcd. for C_17_H_17_FN_7_O_3_S [M + H]^+^ 418.1092, found 418.1194. Retention time 2.79 min, >96% purity.

*Methyl 2-(4-(5-((1-(2-fluoroethyl)-1H-imidazol-4-yl)sulfonyl)-5H-pyrrolo[2,3-b]pyrazin-3-yl)-1H-pyrazol-1-yl)acetate* (**17**). Yield: 75%; white solid. ^1^H-NMR (400 MHz, DMSO-*d*_6_, ppm) δ 8.96 (s, 1H), 8.65 (s, 1H), 8.52 (s, 1H), 8.21 (s, 1H), 8.12 (d, *J* = 4.1 Hz, 1H), 7.87 (s, 1H), 6.95 (d, *J* = 4.1 Hz, 1H), 5.20 (s, 2H), 4.75 (t, *J* = 4.7 Hz, 1H), 4.63 (t, *J* = 4.7 Hz, 1H), 4.44 (t, *J* = 4.6 Hz, 1H), 4.37 (t, *J* = 4.6 Hz, 1H), 3.73 (s, 3H). ESI-MS: C_17_H_17_FN_7_O_4_S, Exact Mass: 433.10, *m*/*z* 434.16 (M + 1)^+^. Retention time 2.61 min, >97% purity.

*1-(4-(5-((1-(2-Fluoroethyl)-1H-imidazol-4-yl)sulfonyl)-5H-pyrrolo[2,3-b]pyrazin-3-yl)-1H-pyrazol-1-yl)propan-2-one* (**18**). Yield: 80%; white solid. ^1^H-NMR (400 MHz, CDCl_3_, ppm) δ 8.72 (s, 1H), 8.08 (s, 1H), 8.07 (s, 1H), 8.07 (s, 1H), 8.03 (d, *J* = 4.2 Hz, 1H), 7.49 (s, 1H), 6.82 (d, *J* = 4.3 Hz, 1H), 5.03 (s, 2H), 4.68 (d, *J* = 4.8 Hz, 1H), 4.56 (d, *J* = 4.4 Hz, 1H), 4.33 (d, *J* = 4.5 Hz, 1H), 4.26 (d, *J* = 4.5 Hz, 1H), 2.24 (s, 3H). ESI-MS: C_17_H_17_FN_7_O_3_S, Exact Mass: 417.10, *m*/*z* 418.13 (M + 1)^+^. HRMS-ESI *m*/*z* calcd. for C_17_H_17_FN_7_O_3_S [M + H]^+^ 418.1092, found 418.1222. Retention time 2.48 min, >96% purity.

*Methyl 2-(4-(5-((1-(2-fluoroethyl)-1H-imidazol-4-yl)sulfonyl)-5H-pyrrolo[2,3-b]pyrazin-3-yl)-1H-pyrazol-1-yl)acetate* (**19**). Yield: 76%; white solid. ^1^H-NMR (400 MHz, DMSO-*d*_6_, ppm) δ 8.96 (s, 1H), 8.65 (s, 1H), 8.52 (s, 1H), 8.21 (s, 1H), 8.12 (d, *J* = 4.1 Hz, 1H), 7.87 (s, 1H), 6.95 (d, *J* = 4.1 Hz, 1H), 5.20 (s, 2H), 4.75 (t, *J* = 4.7 Hz, 1H), 4.63 (t, *J* = 4.7 Hz, 1H), 4.44 (t, *J* = 4.6 Hz, 1H), 4.37 (t, *J* = 4.6 Hz, 1H), 3.73 (s, 3H). ESI-MS: C_17_H_17_FN_7_O_4_S, Exact Mass: 433.10, *m*/*z* 434.16 (M + 1)^+^. HRMS-ESI *m*/*z* calcd. for C_17_H_17_FN_7_O_4_S [M + H]^+^ 434.1041, found 434.1252. Retention time 2.61 min, >97% purity.

*Ethyl 2-(4-(5-((1-(2-fluoroethyl)-1H-imidazol-4-yl)sulfonyl)-5H-pyrrolo[2,3-b]pyrazin-3-yl)-1H-pyrazol-1-yl)acetate* (**20**). Yield: 78%; yellow solid. ^1^H-NMR (400 MHz, DMSO-*d*_6_, ppm) δ 8.95 (s, 1H), 8.63 (d, *J* = 1.3 Hz, 1H), 8.49 (s, 1H), 8.21 (s, 1H), 8.12 (d, *J* = 4.1 Hz, 1H), 7.86 (d, *J* = 1.1 Hz, 1H), 6.94 (d, *J* = 4.1 Hz, 1H), 5.16 (s, 2H), 4.74 (t, *J* = 4.0 Hz, 1H), 4.63 (t, *J* = 4.0 Hz, 1H), 4.43 (t, *J* = 4.0 Hz, 1H), 4.35 (t, *J* = 4.1 Hz, 1H), 4.19 (q, *J* = 7.1 Hz, 2H), 1.24 (t, *J* = 7.1 Hz, 3H). ESI-MS: C_18_H_19_FN_7_O_4_S, Exact Mass: 447.11, *m*/*z* 448.16 (M + 1)^+^. HRMS-ESI *m*/*z* calcd. for C_18_H_19_FN_7_O_4_S [M + H]^+^ 448.1198, found 448.1446. Retention time 2.80 min, >98% purity.

*2-(4-(5-((1-(2-Fluoroethyl)-1H-imidazol-4-yl)sulfonyl)-5H-pyrrolo[2,3-b]pyrazin-3-yl)-1H-pyrazol-1-yl)-N-methylacetamide* (**21**). Yield: 79%; white solid. ^1^H-NMR (400 MHz, DMSO-*d*_6_, ppm) δ 8.96 (s, 1H), 8.65 (s, 1H), 8.46 (s, 1H), 8.18 (s, 1H), 8.12 (d, *J* = 4.1 Hz, 1H), 8.08–8.04 (m, 1H), 7.86 (s, 1H), 6.94 (d, *J* = 4.1 Hz, 1H), 4.86 (s, 2H), 4.76 (t, *J* = 4.6 Hz, 1H), 4.64 (t, *J* = 4.7 Hz, 1H), 4.44 (t, *J* = 4.7 Hz, 1H), 4.37 (t, *J* = 4.7 Hz, 1H), 2.65 (d, *J* = 4.6 Hz, 3H). ESI-MS: C_17_H_18_FN_8_O_3_S, Exact Mass: 432.11, *m*/*z* 433.16 (M + 1)^+^. HRMS-ESI *m*/*z* calcd. for C_17_H_18_FN_8_O_3_S [M + H]^+^ 433.1201, found 433.1226. Retention time 2.36 min, >98% purity.

*2-(4-(5-((1-(2-Fluoroethyl)-1H-imidazol-4-yl)sulfonyl)-5H-pyrrolo[2,3-b]pyrazin-3-yl)-1H-pyrazol-1-yl)-N,N-dimethylacetamide* (**22**). Yield: 81%; yellow solid. ^1^H-NMR (400 MHz, DMSO-*d*_6_, ppm) δ 8.95 (s, 1H), 8.63 (d, *J* = 1.2 Hz, 1H), 8.39 (s, 1H), 8.15 (s, 1H), 8.11 (d, *J* = 4.1 Hz, 1H), 7.86 (d, *J* = 1.1 Hz, 1H), 6.94 (d, *J* = 4.1 Hz, 1H), 5.20 (s, 2H), 4.75(t, *J* = 4.7 Hz, 1H), 4.67(t, *J* = 4.7 Hz, 1H), 4.43 (t, *J* = 4.7 Hz, 1H), 4.36 (t, *J* = 4.7 Hz, 1H), 3.08 (s, 3H), 2.89 (s, 3H). ESI-MS: C_18_H_20_FN_8_O_3_S, Exact Mass: 446.13, *m*/*z* 447.21 (M + 1)^+^. HRMS-ESI *m*/*z* calcd. for C_18_H_20_FN_8_O_3_S [M + H]^+^ 447.1358, found 447.1574. Retention time 2.45 min, >97% purity.

*5-((1-(2-Fluoroethyl)-1H-imidazol-4-yl)sulfonyl)-3-(1-(methylsulfonyl)-1H-pyrazol-4-yl)-5H-pyrrolo[2,3-b]pyrazine* (**23**). Yield: 85%; white solid. ^1^H-NMR (400 MHz, DMSO-*d*_6_, ppm) δ 9.17 (s, 1H), 9.02 (s, 1H), 8.70 (d, *J* = 1.4 Hz, 1H), 8.59 (s, 1H), 8.22 (d, *J* = 4.1 Hz, 1H), 7.87 (d, *J* = 1.2 Hz, 1H), 6.99 (d, *J* = 4.2 Hz, 1H), 4.76 (t, *J* = 4.7 Hz, 1H), 4.65 (t, *J* = 4.7 Hz, 1H), 4.43 (t, *J* = 4.7 Hz, 1H), 4.36 (t, *J* = 4.6 Hz, 1H), 3.64 (s, 3H). ESI-MS: C_15_H_15_FN_7_O_4_S_2_, Exact Mass: 439.05, *m*/*z* 440.14 (M + 1)^+^. HRMS-ESI *m*/*z* calcd. for C_15_H_15_FN_7_O_4_S_2_ [M + H]^+^ 440.0605, found 440.0659. Retention time 2.67 min, >98% purity.

*3-(1-(Cyclopropylsulfonyl)-1H-pyrazol-4-yl)-5-((1-(2-fluoroethyl)-1H-imidazol-4-yl)sulfonyl)-5H-pyrrolo[2,3-b]pyrazine* (**24**). Yield: 82%; white solid. ^1^H-NMR (400 MHz, CDCl_3_, ppm) δ 8.77 (s, 1H), 8.52 (s, 1H), 8.33 (s, 1H), 8.12 (d, *J* = 4.2 Hz, 1H), 8.08 (s, 1H), 7.52 (s, 1H), 6.86 (d, *J* = 4.1 Hz, 1H), 4.72 (t, *J* = 4.6 Hz, 1H), 4.63 (t, *J* = 4.6 Hz, 1H), 4.34 (t, *J* = 4.6 Hz, 1H), 4.26 (t, *J* = 4.6 Hz, 1H), 2.89–2.82 (m, 1H), 1.29–1.24 (m, 2H), 0.86–1.83 (m, 2H). ESI-MS: C_17_H_17_FN_7_O_4_S_2_, Exact Mass: 465.07, *m*/*z* 466.12 (M + 1)^+^. HRMS-ESI *m*/*z* calcd. for C_17_H_17_FN_7_O_4_S_2_ [M + H]^+^ 466.0762, found 466.0780. Retention time 2.92 min, >96% purity.

*Dimethyl 2-(4-(5-((1-(2-fluoroethyl)-1H-imidazol-4-yl)sulfonyl)-5H-pyrrolo[2,3-b]pyrazin-3-yl)-1H-pyrazol-1-yl)malonate* (**25**). Yield: 82%; yellow solid. ^1^H-NMR (400 MHz, CDCl_3_, ppm) δ 8.74 (s, 1H), 8.38 (s, 1H), 8.12 (s, 1H), 8.08 (s, 1H), 8.05 (d, *J* = 4.4 Hz, 1H), 7.50 (s, 1H), 6.82 (d, *J* = 4.0 Hz, 1H), 5.96 (s, 1H), 4.67(t, *J* = 4.5 Hz, 1H), 4.56 (t, *J* = 4.5 Hz, 1H), 4.34 (t, *J* = 4.4Hz, 1H), 4.27 (t, *J* = 4.5 Hz, 1H), 3.90 (s, 6H). ^13^C-NMR (126 MHz, CD_3_OD, ppm) δ 165.08, 141.71, 140.11, 139.04, 138.26, 137.36, 136.82, 130.59, 130.30, 127.91, 121.62, 105.14, 82.54, 81.20, 52.77, 52.72, 29.36, 28.92, 13.02. ESI-MS: C_19_H_19_FN_7_O_6_S, Exact Mass: 491.10, *m*/*z* 492.18 (M + 1)^+^. HRMS-ESI *m*/*z* calcd. for C_19_H_19_FN_7_O_6_S [M + H]^+^ 492.1096, found 492.1241. Retention time 2.79 min, >98% purity.

*Diethyl 2-(4-(5-((1-(2-fluoroethyl)-1H-imidazol-4-yl)sulfonyl)-5H-pyrrolo[2,3-b]pyrazin-3-yl)-1H-pyrazol-1-yl)malonate* (**26**). Yield: 76%; white solid. ^1^H-NMR (400 MHz, CDCl_3_, ppm) δ 8.73 (s, 1H), 8.39 (s, 1H), 8.12 (s, 1H), 8.08 (s, 1H), 8.04 (d, *J* = 4.1 Hz, 1H), 7.50 (s, 1H), 6.81 (d, *J* = 4.1 Hz, 1H), 5.91 (s, 1H), 4.66 (t, *J* = 4.5 Hz, 1H), 4.55 (t, *J* = 4.5 Hz, 1H), 4.40–4.30 (m, 5H), 4.27(t, *J* = 4.5 Hz, 1H), 1.36 (t, *J* = 7.1 Hz, 6H). ESI-MS: C_21_H_23_FN_7_O_6_S, Exact Mass: 519.13, *m*/*z* 520.24 (M + 1)^+^. HRMS-ESI *m*/*z* calcd. for C_21_H_23_FN_7_O_6_S [M + H]^+^ 520.1409, found 520.1667. Retention time 3.11 min, >92% purity.

General Procedure for the Synthesis of *3-bromo-5-((1-ethyl-1H-imidazol-4-yl)sulfonyl)-5H-pyrrolo[2,3-b]pyrazine* (**46**). NaH (130 mg, 5.52 mmol) was suspended in dry DMF. 5-((1*H*-imidazol-4-yl)sulfonyl)-3-bromo-5*H*-pyrrolo[2,3-*b*]pyrazine (**42**, 1 g, 3 mmol) and iodoethane (562 mg, 3.6 mmol) were dissolved in the DMF. Then, the resulting mixture was stirred at 80 °C for 4 h. After the reaction was cooled to the room temperature, water (30 mL) was added to the reaction system. The aqueous layer was extracted with EtOAc (2 × 20 mL). The combined organic layers were dried (Na_2_SO_4_), filtered, and concentrated to afford compound **46** as a yellow hairy solid with the yield of 82%. ^1^H-NMR (400 MHz, CDCl_3_, ppm) δ 8.57 (s, 1H), 8.09–8.04 (m, 2H), 7.48 (d, *J* = 1.1 Hz, 1H), 6.82 (d, *J* = 4.1 Hz, 1H), 4.08 (q, *J* = 7.3 Hz, 2H), 1.53 (t, *J* = 7.4 Hz, 3H). ESI-MS: C_11_H_11_BrN_5_O_2_S, Exact Mass: 354.97, *m*/*z* 356.03 (M + 1)^+^. Retention time 2.80 min, >98% purity.

General Procedure for the Synthesis of *5-((1-ethyl-1H-imidazol-4-yl)sulfonyl)-3-(1H-pyrazol-4-yl)-5H-pyrrolo[2,3-b]pyrazine* (**29**). 3-bromo-5-((1-ethyl-1*H*-imidazol-4-yl)sulfonyl)-5*H*-pyrrolo[2,3-*b*]pyrazine (**46**, 50 mg, 0.14 mmol), 4-(4,4,5,5-tetramethyl-1,3,2-dioxaborolan-2-yl)-1*H*-pyrazole (33 mg, 0.17 mmol), K_2_CO_3_ (58 mg, 0.42 mmol) and Pd(dppf)Cl_2_ (6 mg, 0.007 mmol) was dissolved in the solvent of 1,4-dioxane/H_2_O (*v*/*v* = 3:1, 20 mL). The resulting mixture was stirred at 80 °C under Ar for 4 h. Then, the reaction mixture was evaporated to dryness. The residue was purified by flash chromatography to give compound **29** as a yellow hairy solid with the yield of 87%. ^1^H-NMR (400 MHz, DMSO-*d*_6_, ppm) δ 13.25 (s, 1H), 8.97 (s, 1H), 8.73 (s, 1H), 8.52 (s, 1H), 8.21 (s, 1H), 8.09 (d, *J* = 4.1 Hz, 1H), 7.84 (s, 1H), 6.92 (d, *J* = 4.1 Hz, 1H), 4.06 (q, *J* = 7.3 Hz, 2H), 1.30 (t, *J* = 7.2 Hz, 3H). ESI-MS: C_14_H_14_N_7_O_2_S, Exact Mass: 343.09, *m*/*z* 344.11 (M + 1)^+^. HRMS-ESI *m*/*z* calcd. for C_14_H_14_N_7_O_2_S [M + H]^+^ 344.0924, found 344.0929. Retention time 2.34 min, >98% purity.

The compounds **27**–**33** were prepared as described for the synthesis of compound **29** (Scheme 3).

*3-((1H-Imidazol-4-yl)sulfonyl)-3-bromo-5H-pyrrolo[2,3-b]pyrazine* (**44**). Yield: 79%; white solid. ^1^H-NMR (400 MHz, CD_3_OD, ppm) δ 8.62 (s, 1H), 8.22 (d, *J* = 1.1 Hz, 1H), 8.16 (d, *J* = 4.2 Hz, 1H), 7.79 (d, *J* = 1.0 Hz, 1H), 6.88 (d, *J* = 4.1 Hz, 1H). ESI-MS: C_9_H_7_BrN_5_O_2_S, Exact Mass: 326.94, *m*/*z* 328.11 (M + 1)^+^. Retention time 2.40 min, >98% purity.

*5-((1H-Imidazol-4-yl)sulfonyl)-3-(1H-pyrazol-4-yl)-5H-pyrrolo[2,3-b]pyrazine* (**27**). Yield: 80%; white solid. ^1^H-NMR (400 MHz, DMSO-*d*_6_, ppm) δ 13.24 (s, 1H), 13.14 (s, 1H), 8.96 (s, 1H), 8.57 (s, 1H), 8.50 (s, 1H), 8.17 (s, 1H), 8.10 (d, *J* = 4.1 Hz, 1H), 7.82 (s, 1H), 6.91 (d, *J* = 4.1 Hz, 1H). ESI-MS: C_12_H_10_N_7_O_2_S, Exact Mass: 315.05, *m*/*z* 316.09(M + 1)^+^. HRMS-ESI *m*/*z* calcd. for C_12_H_10_N_7_O_2_S [M + H]^+^ 316.0611, found 316.0607. Retention time 2.18 min, >98% purity.

*3-Bromo-5-((1-methyl-1H-imidazol-4-yl)sulfonyl)-5H-pyrrolo[2,3-b]pyrazine* (**45**). Yield: 76%; white solid. ^1^H-NMR (400 MHz, CDCl_3_, ppm) δ 8.58 (s, 1H), 8.07 (d, *J* = 4.1 Hz, 1H), 7.99 (d, *J* = 1.4 Hz, 1H), 7.43 (s, 1H), 6.82 (d, *J* = 4.1 Hz, 1H), 3.79 (s, 3H). ESI-MS: C_10_H_9_BrN_5_O_2_S, Exact Mass: 340.96, *m*/*z* 341.91 (M + 1)^+^. Retention time 2.23 min, >98% purity.

*5-((1-Methyl-1H-imidazol-4-yl)sulfonyl)-3-(1H-pyrazol-4-yl)-5H-pyrrolo[2,3-b]pyrazine* (**28**). Yield: 82%; yellow solid. ^1^H-NMR (400 MHz, DMSO-*d*_6_, ppm) δ 13.25 (s, 1H), 8.97 (s, 1H), 8.59 (s, 1H), 8.50 (s, 1H), 8.20 (s, 1H), 8.08 (d, *J* = 4.1 Hz, 1H), 7.77 (s, 1H), 6.92 (d, *J* = 4.1 Hz, 1H), 3.71 (s, 3H). ^13^C-NMR (126 MHz, DMSO-*d*_6_, ppm) δ 142.75, 141.24, 140.13, 138.69, 138.65, 138.01, 135.91, 130.55, 129.73, 128.19, 119.70, 106.08, 34.28. ESI-MS: C_13_H_12_N_7_O_2_S, Exact Mass: 329.07, *m*/*z* 330.08 (M + 1)^+^. HRMS-ESI *m*/*z* calcd. for C_13_H_12_N_7_O_2_S [M + H]^+^ 330.0768, found 330.0771. Retention time 2.25 min, >96% purity.

*3-Bromo-5-((1-isopropyl-1H-imidazol-4-yl)sulfonyl)-5H-pyrrolo[2,3-b]pyrazine* (**47**). Yield: 83%; white solid. ^1^H-NMR (400 MHz, CDCl_3_, ppm) δ 8.57 (s, 1H), 8.15 (d, *J* = 1.3 Hz, 1H), 8.08 (d, *J* = 4.1 Hz, 1H), 7.53 (d, *J* = 1.2 Hz, 1H), 6.82 (d, *J* = 4.1 Hz, 1H), 4.48–4.36 (m, 1H), 1.55 (d, *J* = 6.7 Hz, 6H). ESI-MS: C_12_H_13_BrN_5_O_2_S, Exact Mass: 368.99, *m*/*z* 370.06 (M + 1)^+^. Retention time 2.96 min, >98% purity.

*6-((1-Isopropyl-1H-imidazol-4-yl)sulfonyl)-3-(1H-pyrazol-4-yl)-5H-pyrrolo[2,3-b]pyrazine* (**30**). Yield: 80%; white solid. ^1^H-NMR (400 MHz, CDCl_3_, ppm) δ 8.70 (s, 1H), 8.16 (s, 2H), 8.07 (s, 1H), 8.01 (d, *J* = 4.1 Hz, 1H), 7.52 (s, 1H), 6.79 (d, *J* = 4.1 Hz, 1H), 4.41–4.30 (m, 1H), 1.44 (d, *J* = 6.7 Hz, 6H). ^13^C-NMR (126 MHz, CD_3_OD, ppm) δ 142.50, 140.37, 138.60, 138.28, 137.30, 136.41, 130.28, 127.53, 125.78, 119.92, 104.99, 50.90, 22.11(C×2), one signal missing. ESI-MS: C_15_H_16_N_7_O_2_S, Exact Mass: 357.10, *m*/*z* 358.15(M + 1)^+^. HRMS-ESI *m*/*z* calcd. for C_15_H_16_N_7_O_2_S [M + H]^+^ 358.1081, found 358.1081. Retention time 2.42 min, >99% purity.

*2-Bromo-5-((1-isobutyl-1H-imidazol-4-yl)sulfonyl)-5H-pyrrolo[2,3-b]pyrazine* (**48**). Yield: 76%; white solid. ^1^H-NMR (400 MHz, CDCl_3_, ppm) δ 8.57 (s, 1H), 8.08 (d, *J* = 4.1 Hz, 1H), 8.03 (s, 1H), 7.42 (s, 1H), 6.82 (d, *J* = 4.1 Hz, 1H), 3.82 (d, *J* = 7.1 Hz, 2H), 2.12–2.03 (m, 1H), 0.93 (d, *J* = 7.0 Hz, 6H). ESI-MS: C_13_H_15_BrN_5_O_2_S, Exact Mass: 383.01, *m*/*z* 406.1 (M + Na)^+^. Retention time 3.31 min, >96% purity.

*5-((1-Isobutyl-1H-imidazol-4-yl)sulfonyl)-3-(1H-pyrazol-4-yl)-5H-pyrrolo[2,3-b]pyrazine* (**31**). Yield: 79%; white solid. ^1^H-NMR (400 MHz, CDCl_3_, ppm) δ 8.76 (s, 1H), 8.60 (s, 1H), 8.25 (s, 1H), 8.10 (d, *J* = 4.1 Hz, 1H), 7.94 (d, *J* = 1.3 Hz, 1H), 7.39 (d, *J* = 1.2 Hz, 1H), 6.84 (d, *J* = 4.1 Hz, 1H), 3.76 (d, *J* = 7.2 Hz, 2H), 2.06–1.93 (m, 1H), 0.83 (d, *J* = 6.7 Hz, 6H). ESI-MS: C_16_H_18_N_7_O_2_S, Exact Mass: 371.12, *m*/*z* 372.16 (M + 1)^+^. HRMS-ESI *m*/*z* calcd. for C_16_H_18_N_7_O_2_S [M + H]^+^ 372.1237, found 372.1281. Retention time 3.35 min, >97% purity.

*3-Bromo-5-((1-cyclopentyl-1H-imidazol-4-yl)sulfonyl)-5H-pyrrolo[2,3-b]pyrazine* (**49**). Yield: 82%; white solid. ^1^H-NMR (400 MHz, CDCl_3_, ppm) δ 8.57 (s, 1H), 8.11 (d, *J* = 1.3 Hz, 1H), 8.07 (d, *J* = 4.1 Hz, 1H), 7.51 (d, *J* = 1.3 Hz, 1H), 6.81 (d, *J* = 4.1 Hz, 1H), 4.57–4.49 (m, 1H), 2.30–2.21 (m, 2H), 1.95–1.77 (m, 6H). ESI-MS: C_14_H_15_BrN_5_O_2_S, Exact Mass: 395.01, *m*/*z* (M + 1)^+^. Retention time 2.35 min, >98% purity.

*5-((1-Cyclopentyl-1H-imidazol-4-yl)sulfonyl)-3-(1H-pyrazol-4-yl)-5H-pyrrolo[2,3-b]pyrazine* (**32**). Yield: 86%; white solid. ^1^H-NMR (400 MHz, CDCl_3_, ppm) δ 8.74 (s, 1H), 8.18 (s, 2H), 8.03 (d, *J* = 4.1 Hz, 1H), 8.01 (s, 1H), 7.49 (s, 1H), 6.81 (d, *J* = 4.1 Hz, 1H), 4.51–4.43 (m, 1H), 2.19 (td, *J* = 14.9, 8.5 Hz, 4H), 1.77–1.65 (m, 4H). ESI-MS: C_17_H_18_N_7_O_2_S, Exact Mass: 383.12, *m*/*z* 384.15 (M + 1)^+^. HRMS-ESI *m*/*z* calcd. for C_17_H_18_N_7_O_2_S [M + H]^+^ 384.1237, found 384.1395. Retention time 2.60 min, >96% purity.

*2-(4-((3-(1H-Pyrazol-4-yl)-5H-pyrrolo[2,3-b]pyrazin-5-yl)sulfonyl)-1H-imidazol-1-yl)-N-methylacetamide* (**33**). Yield: 84%; white solid. ^1^H-NMR (400 MHz, DMSO-*d*_6_, ppm) δ 13.22 (s, 1H), 8.97 (s, 1H), 8.52 (s, 1H), 8.49 (s, 1H), 8.18 (s, 1H), 8.14–8.08 (m, 2H), 7.75 (s, 1H), 6.92 (d, *J* = 4.1 Hz, 1H), 4.77 (s, 2H), 2.57 (d, *J* = 4.5 Hz, 3H). ESI-MS: C_15_H_15_N_8_O_3_S, Exact Mass: 386.09, *m*/*z* 387.14(M + 1)^+^. HRMS-ESI *m*/*z* calcd. for C_15_H_15_N_8_O_3_S [M + H]^+^ 387.0982, found 387.1067. Retention time 2.57 min, >98% purity.

### 3.2. Crystallography and Docking Study

Production of the kinase domain（residues 458–756, with mutagenesis of C488A and C584S）of recombinant human FGFR1 followed the protocols of Mohammadi et al. [28] with certain modifications. The cDNA fragment was cloned into the modified pET28a vector, in which a TEV protease site was inserted at the multiple cloning site. The kinase domain of FGFR1 was co-expressed with catYopH subcloned in pET15b (164-468AA) The expressed protein was passed through a Ni-NTA column (Qiagen). After TEV protease incubation, the kinase domain was further purified by the Q HP ion exchange column (GE), which eluted with 20 mM Tris pH 7.5, 100 mM NaCl, 10% glycerol, 1 mM DTT. The eluted protein was buffer exchanged into 20 mM Tris-HCl pH 8.0, 20 mM NaCl, 2 mM DTT using Hiprep 16/60 Superdex 75 column (GE health). The protein was concentrated to ~12 mg/mL for further crystallization.

Crystallization of the FGFR1 kinase domain was carried out by mixing a solution of the protein with an equal volume of precipitant solution (0.1 M bis-Tris pH 6.5, 0.3 M (NH_4_)_2_SO_4_, 5% Glycerol, 15–20% PEG10K). Crystallization utilized the vapor diffusion method in hanging drops at 4 °C. Crystals appeared in a week. The crystal of the protein–ligand complex was obtained by soaking the apo crystals in a buffer (0.1 M bis-Tris pH 6.5, 0.3 M (NH_4_)_2_SO_4_, 5% Glycerol, 25% PEG10K) containing 1 mM inhibitor. Crystals of the complex were then flash frozen in liquid nitrogen in the presence of a soaking buffer.

Data were collected at 100 K at the Shanghai Synchrotron Radiation Facility (SSRF), and were processed with the XDS4 software packages. The structure was solved by molecular replacement, using the program PHASER5 with the search model of pdb code 1AGW6. The structure was refined with the simulated annealing protocol implemented in the program PHENIX7. With the aid of the program Coot8, the compound, as well as water molecules, were fitted into to the initial Fo–Fc map.

Docking studies of inhibitor **13** were performed using Glide (Schrodinger, LLC) with the Extra-Precision (XP) scoring function. The ligands in the docking study were prepared using LigPrep (Schrödinger, LLC). The binding region was defined by a 30 Å × 30 Å × 30 Å grid box centered on the binding site of the 5Z0S (compound 8 bound to the FGFR1 structure). Default parameter settings were used in the Glide docking simulation.

### 3.3. ELISA Kinase Assay

The effects of compounds on the activities of indicated (FGFR1 and c-Met) kinases were determined using enzyme-linked immunosorbent assays (ELISAs) with purified recombinant proteins. Briefly, 20 μg/mL poly (Glu, Tyr)_4:1_ (Sigma, St. Louis, MO, USA) was precoated in 96-well plates as a substrate. A 50-μL aliquot of 10 μmol/L ATP solution diluted in kinase reaction buffer (50 mmol/L HEPES (pH 7.4), 50 mmol/L MgCl_2_, 0.5 mmol/L MnCl_2_, 0.2 mmol/L Na_3_VO_4_, and 1 mmol/L DTT) was added to each well; 1 μL of various concentrations of compounds diluted in 1% DMSO (*v*/*v*) (Sigma) were then added to each reaction well. DMSO (1%, *v*/*v*) was used as the negative control. The kinase reaction was initiated by the addition of purified tyrosine kinase proteins FGFR1 (Millipore, Darmstadt, Germany) or c-Met (Millipore) diluted in 49 μL of kinase reaction buffer. After incubation for 60 min at 37 °C, the plate was washed three times with phosphate-buffered saline (PBS) containing 0.1% Tween 20 (T-PBS). Anti-phosphotyrosine (PY99) antibody (Sanra Cruz, Dallas, TX, USA) (100 μL; 1:500, diluted in 5 mg/mL bovine serum albumin (BSA) T-PBS) was then added. After a 30-min incubation at 37 °C, the plate was washed three times, and 100 μL of horseradish peroxidase-conjugated goat anti-mouse IgG (Calbiochem, Shanghai, China) (1:2000, diluted in 5 mg/mL BSA T-PBS) was added. The plate was then incubated at 37 °C for 30 min, and washed three times. A 100-μL aliquot of a solution containing 0.03% H_2_O_2_ and 2 mg/mL o-phenylenediamine in 0.1 mol/L citrate buffer (pH 5.5) was added. The reaction was terminated by the addition of 50 μL of 2 mol/L H_2_SO_4_ as the color changed, and the plate was analyzed using a multi-well spectrophotometer (SpectraMAX 190, Molecular Devices, Palo Alto, CA, USA) at 490 nm. The inhibition rate (%) was calculated using the following equation:

IR = [1 − (A_490_/A_490_control)] × 100%. (1)

where IR, A_490_, and A_490_control are the inhibition rate, the absorbance value of the tested compound at 490 nm, and the absorbance value of the negative control compound at 490 nm, respectively. The IC_50_ values were calculated from the inhibition curves in two separate experiments.

### 3.4. Cell Proliferation Assay

Cell proliferation was examined in a FGFR1-translocated KG1 leukemia cell line harboring FGFR1OP-FGFR1 fusion. The original KG1 cell line was purchased from ATCC (CRL-8031), and cultured according the ATCC standard protocol. Cells were seeded in 96-well tissue culture plates. On the next day, the cells were exposed to various concentrations of compounds and further cultured for 72 h. Cell proliferation was then determined using sulforhodamine B (SRB, from Sigma-Aldrich, St Louis, MO, USA) or the thiazolyl blue tetrazolium bromide (MTT, from Sigma-Aldrich, St Louis, MO, USA) assay. The IC50 values were calculated by concentration- response curve fitting using the four-parameter method.

### 3.5. In Vitro Metabolic Stability Study

Microsomes (Human microsome: Xenotech, Lot No.H0610; Rat microsome: Xenotech, Lot No. R1000) (0.5 mg/mL) were preincubated with 1 μM of test compound for 5 min at 37 °C in 0.1 M phosphate buffer (pH 7.4) with 1 mmol Ethylenediaminetetraacetic acid (EDTA), and 5 mmol MgCl_2_. The reactions were initiated by adding prewarmed cofactors (1 mmol NADPH). After 0-min, 5-min, 10-min, and 30-min incubations at 37 °C, the reactions were stopped by adding an equal volume of cold acetonitrile. The samples were vortexed for 10 min, and then centrifuged at 10,000× *g* for 10 min. Supernatants were analyzed by LC/MS/MS for the amount of the parent compound remaining, and the corresponding loss of the parent compound was also determined by LC/MS/MS. 

The Cytochromes P450 (CYP) enzymatic activities were characterized based on their probe reactions: CYP3A4 (midazolam), CYP2D6 (dextromethorphan), CYP2C9 (Diclofenac), CYP1A2 (phenacetin) and CYP2C19 (mephenytoin). Incubation mixtures were prepared in a total volume of 100 μL as follows: 0.2 mg/mL of microsome (Human microsome: Xenotech, Lot No.H0610), 1 mmol of NADPH, 100 mmol of phosphate buffer (pH 7.4), probe substrates cocktail (10 μM of midazolam, 100 μM of testosterone, 10 μM of dextromethophan, 20 μM of diclofenac, 100 μM of phenacetin, 100 μM of mephenytoin), and 10 μM of the tested compound or positive control cocktail (10 μM of ketoconazole, 10 μM of quinidine，100 μM of sulfaphenazole，10 μM of naphthoflavone, and 1000 μM of tranylcypromine) or negative control (PBS). The final concentration of the organic reagent in the incubation mixtures was less than 1% *v*/*v*. There was a 5-min preincubation period at 37 °C before the reaction was initiated by adding a nicotinamide adenine dinucleotide phosphate (NADPH)-generating system. Reactions were conducted for 20 min for CYPs. For each probe drug, the percentage of the metabolite conversion was less than 20% of the substrate added. The inhibition rate was calculated as: (the formation of the metabolite of probe substrates with 10 μM of the tested compound)/(the formation of the metabolite of the probe substrates with PBS) × 100%. 

## 4. Conclusions

Starting with the scaffold of 1-sulfonylpyrazolo[4,3-*b*]pyridine discovered in c-Met inhibitor development, we developed a series of new pyrrolo[2,3-*b*]pyrazine analogues. Based off of the X-ray crystal structure of compound **8** bound with FGFR1, we found that it showed novel binding conformation, which significantly differed to c-Met ligand binding. Extensive structure–activity relationships were conducted, and compound **13** was identified as possessing high enzymatic and cellular potency against FGFR1. The activity difference between FGFR and c-Met indicated that the series of inhibitors might be selective FGFR inhibitors. Moreover, through in vitro metabolic stability study, compound **13** shown favorable metabolic properties. All of these data indicated that compound **13** would be a promising lead compound for further drug development.

## Figures and Tables

**Table 1:** The FGFR1 enzymatic activity of compounds **5**–**8**. ^a^

Compound	Structure	FGFR1 Inhibition (%)
		10 μM
**5**	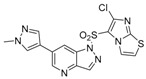	64.4
**6**	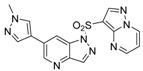	80.1
**7**	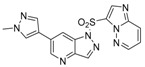	86.9
**8**	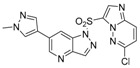	89.7

^a^ Inhibition ratio are given as the mean from two separate experiments.

**Table 2:** The FGFR1 enzymatic activity of compounds **9**–**12**. ^a^

Compound	Structure	FGFR1 Inhibition (%)	
		10 μM	5 μM
**9**	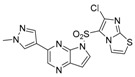	92.5	83.7
**10**	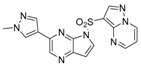	91.0	77.0
**11**	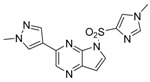	86.0	58.1
**12**	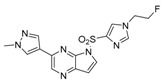	89.0	68.9

^a^ Inhibition ratio are given as the mean from two separate experiments.

**Table 3:**
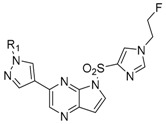
The FGFR1 enzymatic activity of compounds **13**–**26**. ^a^

Compound	R_1_	FGFR1 Inhibition (%)		
		1 μM	0.1 μM	0.01 μM
**13**	H	100.5	91.7	93.3
**14**	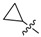	2.8	2.2	4.7
**15**	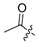	70.9	25.9	35.9
**16**	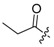	94.6	55.8	15.1
**17**	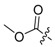	56.8	15.6	−3.3
**18**	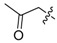	22.6	13.3	24.6
**19**	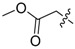	40.7	12.0	7.3
**20**	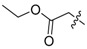	49.6	5.4	7.2
**21**	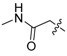	65.6	14.9	12.5
**22**	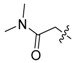	15.3	2.5	0.4
**23**	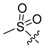	82.3	37.1	20.9
**24**	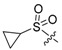	29.5	13.5	16.6
**25**	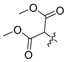	83.0	46.5	23.5
**26**	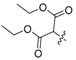	69.0	33.6	26.8

^a^ Inhibition ratio are given as the mean from two separate experiments.

**Table 4:**
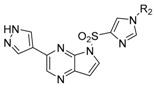
The FGFR1 enzymatic activity of compounds **27**–**35**. ^a^

Compound	R_2_	FGFR1 Inhibition (%)		
		1 μM	0.1 μM	0.01 μM
**27**	H	90.7	71.9	27.9
**28**	-CH_3_	88.8	63.2	12.5
**29**	-C_2_H_5_	94.6	79.5	53.7
**30**	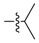	94.5	77.1	40.4
**31**	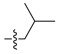	37.1	19.9	13.9
**32**	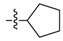	71.7	20.1	4.9
**33**	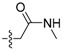	82.4	43.8	19.9
**34**	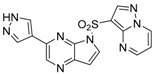	73.9	26.4	-
**35**	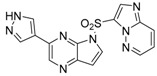	90.6	68.7	31.8

^a^ Inhibition ratios are given as the mean from two separate experiments.

**Table 5:** The IC_50_ of FGFR1 enzymatic activity and KG1 antiproliferation activity of compounds **13, 27**–**29**. ^a^

Cpd.	FGFR1	KG1
	IC_50_ (nM)	IC_50_ (nM)
**13**	0.6 ± 0.2	29.1 ± 5.5
**27**	15.7 ± 4.8	187.1 ± 17.2
**28**	15.4 ± 3.0	98.5 ± 19.1
**29**	3.0 ± 0.7	17.4 ± 8.7

^a^ IC_50_ values are given as the mean ± SD (nM) from two separate experiments.

**Table 6:** The kinase panel profiling of compound **13**. ^a^

Concentration		Kinase Inhibition（%）								
		FGFR2	FGFR3	c-Met	KDR	VEGFR-1	PDGFR-α	PDGFR-β	EGFR	ErbB2
**13**	1000 nM	97.2	88.9	4.6	46.3	0.0	23.2	58.6	0.0	0.0
	100 nM	92.0	79.0	3.9	17.1	4.9	20.9	30.1	0.0	0.0
	10 nM	60.9	42.9	3.9	7.4	0.0	21.9	12.8	0.0	0.0
		**ErbB4**	**Src**	**Abl**	**EPH-A2**	**IGF1R**	**ROS1**	**AXL**	**DDR2**	
	1000 nM	3.0	0.0	0.0	0.0	11.8	11.8	−5.5	4.6	
	100 nM	0.0	0.0	0.0	0.0	3.0	3.0	2.9	0.9	
	10 nM	0.0	0.0	0.0	0.0	−3.8	−3.8	1.1	3.6	

^a^ Inhibition ratio are given as the mean from two separate experiments.

**Table 7:** The in vitro metabolic stability test of compound **13**.

Compound	HLM ^a^	CYPs Direct Inhibition					TDI ^b^
		3A4	2D6	2C9	1A2	2C19	
**13**	1	37%	25%	NI	6%	24%	NI ^c^

^a^ human liver microsome stability test, unit: μL/min/mg. ^b^ TDI means time-dependent inhibition. ^c^ NI means no inhibition.
